# Long-term association of air pollution and incidence of lung cancer among older Americans: A national study in the Medicare cohort

**DOI:** 10.1016/j.envint.2023.108266

**Published:** 2023-10-14

**Authors:** Cristina Su Liu, Yaguang Wei, Mahdieh Danesh Yazdi, Xinye Qiu, Edgar Castro, Qiao Zhu, Longxiang Li, Petros Koutrakis, Christine C. Ekenga, Liuhua Shi, Joel D. Schwartz

**Affiliations:** a Department of Environmental Health, Harvard T.H. Chan School of Public Health, 655 Huntington Ave, Boston, MA 02115, USA; b Program in Public Health, Department of Family, Population and Preventive Medicine, Stony Brook University, 101 Nicolls Road Health Sciences Center, Stony Brook, NY 11794, USA; c Gangarosa Department of Environmental Health, Rollins School of Public Health, Emory University, 1518 Clifton Rd. NE, Atlanta, GA 30322, USA; d Department of Epidemiology, Harvard T.H. Chan School of Public Health, 655 Huntington Ave, Boston, MA 02115, USA

**Keywords:** Air pollution, Lung cancer, Incidence, Medicare

## Abstract

**Background:**

Despite strong evidence of the association of fine particulate matter (PM_2.5_) exposure with an increased risk of lung cancer mortality, few studies had investigated associations of multiple pollutants simultaneously, or with incidence, or using causal methods. Disparities were also understudied.

**Objectives:**

We investigated long-term effects of PM_2.5_, nitrogen dioxide (NO_2_), warm-season ozone, and particle radioactivity (PR) exposures on lung cancer incidence in a nationwide cohort.

**Methods:**

We conducted a cohort study with Medicare beneficiaries (aged ≥ 65 years) continuously enrolled in the fee-for-service program in the contiguous US from 2001 to 2016. Air pollution exposure was averaged across three years and assigned based on ZIP code of residence. We fitted Cox proportional hazards models to estimate the hazard ratio (HR) for lung cancer incidence, adjusted for individual- and neighborhood-level confounders. As a sensitivity analysis, we evaluated the causal relationships using inverse probability weights. We further assessed effect modifications by individual- and neighborhood-level covariates.

**Results:**

We identified 166,860 lung cancer cases of 12,429,951 studied beneficiaries. In the multi-pollutant model, PM_2.5_ and NO_2_ exposures were statistically significantly associated with increased lung cancer incidence, while PR was marginally significantly associated. Specifically, the HR was 1.008 (95% confidence interval [CI]: 1.005, 1.011) per 1-μg/m^3^ increase in PM_2.5_, 1.013 (95% CI: 1.012, 1.013) per 1-ppb increase in NO_2_, and 1.005 (0.999, 1.012) per 1-mBq/m^3^ increase in PR. At low exposure levels, all pollutants were associated with increased lung cancer incidence. Men, older individuals, Blacks, and residents of low-income neighborhoods experienced larger effects of PM_2.5_ and PR.

**Discussion:**

Long-term PM_2.5_, NO_2_, and PR exposures were independently associated with increased lung cancer incidence among the national elderly population. Low-exposure analysis indicated that current national standards for PM_2.5_ and NO_2_ were not restrictive enough to protect public health, underscoring the need for more stringent air quality regulations.

## 1. Introduction

Lung cancer is the leading cause of cancer death and the second most commonly diagnosed cancer worldwide. (Sung et al., 2021) In 2020, there were an estimated 1.8 million deaths and 2.2 million new cases from lung cancer globally. (Chaitanya Thandra et al., 2021) In the United States, lung cancer has the highest mortality rate among all cancers, accounting for approximately 138 thousand deaths (23 % of all cancer deaths) in 2020. (Bade and Dela Cruz, 2020) Cigarette smoking remains the main risk factor for lung cancer. As for other risk factors, the number of lung cancer deaths attributable to ambient fine particulate matter (PM_2.5_) was 0.3 million in 2019 worldwide, contributing to about 15 % of total lung cancer deaths, second to only smoking (63 %). (Yang et al., 2022) Moreover, it was estimated that the number of lung cancer deaths attributable to ambient PM_2.5_ has more than doubled from 1990 to 2019, due to the rapid increase and aging of the global population. In 2013, the International Agency for Research on Cancer (IARC) classified outdoor particulate matter and overall air pollution as Group 1 human carcinogens as a response to increasing scientific evidence of the association between ambient PM_2.5_ and lung cancer (Loomis et al., 2013).

Several epidemiological studies have investigated the impacts of PM_2.5_ on lung cancer mortality, and, less commonly, on lung cancer incidence. A *meta*-analysis conducted by Hamra Ghassan et al (2014) in 2014 found a positive association between PM_2.5_ and lung cancer mortality and incidence among 18 studies. Since then, several other studies have confirmed this finding (Hvidtfeldt et al., 2021; Coleman et al., 2020; Pope et al., 2020; Pun et al., 2017; Bai et al., 2020). In 2015, Hamra et al. (Hamra Ghassan et al., 2015) conducted a second meta-analysis investigating the effect of nitrogen dioxide (NO_2_) on lung cancer mortality and incidence and found a statistically significant increase in risk among 20 studies. Very few studies have assessed the relationship between ozone and lung cancer, with some studies reporting a statistically significant increase in risk (Kazemiparkouhi et al., 2020; Gharibvand et al., 2017) but not others (Hvidtfeldt et al., 2021; Bai et al., 2020; Hystad et al., 2013). The radiometric composition of particulate matter, emitted from the progeny of radon–another leading cause of lung cancer–has been linked to decreased lung function (Nyhan et al., 2019). Nonetheless, the relationship between particle radioactivity (PR) and lung cancer is yet to be studied.

In the US in particular, there is a lack of large-cohort studies that have investigated lung cancer incidence and multiple air pollutants simultaneously. To our knowledge, a study of the Nurses’ Health Study (NHS) cohort was the only large national cohort to investigate lung cancer incidence (Puett Robin et al., 2014), and they found an insignificant increase in the risk of lung cancer associated with PM_2.5_. That study investigated only particulate matter and distance to road as exposures, without considering other pollutants such as NO_2_ and ozone. The study also did not examine health disparities. Further, prior research has predominantly focused on associational relationships, leaving a gap in causal evidence linking air pollution to lung cancer.

To address these knowledge gaps, we investigated the long-term impacts of PM_2.5_, NO_2_, warm-season (i.e., May to October) ozone, and PR exposures on the incidence of lung cancer in a large national cohort of the elderly population enrolled in Medicare across the contiguous US from 2001 to 2016, using a Cox proportional hazards model. As a sensitivity analysis, we evaluated the causal effects using inverse probability weights. Additionally, we evaluated effect measure modifications by individual- and neighborhood-level covariates to identify subpopulations that may experience higher risk.

## 2. Methods

### 2.1. Study population

The study population consisted of nationwide Medicare beneficiaries who were 65 years or older and who lived in the contiguous US between 2001 and 2016. The data were drawn from the Medicare denominator file and the Medicare Chronic Conditions Warehouse (CCW), both from the Centers for Medicare and Medicaid Services (CMS). The denominator file contained enrollment records for all Medicare beneficiaries, including age, sex, Medicaid eligibility, date of death (if applicable), and ZIP code of residence, updated annually. The CCW dataset included the date of the first occurrence of lung cancer diagnosis across all Medicare claims, including physician visits. The study cohort was constructed based on these two Medicare databases with inclusion criteria of continuous enrollment in (1) the Medicare Fee-for-Service program, and (2) both Medicare Part A (hospital insurance) and Part B (medical insurance) over the study period. These criteria were used to exclude beneficiaries who spent any time in Part C (Medicare Advantage Plans) during the study period because claim records for this group of beneficiaries were not available. Also, the enrollment in both Part A and Part B minimized the possibility of missing lung cancer claims, which could happen if a subject was enrolled in only Part A or Part B.

To increase the rigor in identifying lung cancer incidence, we further required a 3-year lung cancer diagnosis-free period after enrollment in Medicare. (Ma et al., 2022) If a beneficiary had been diagnosed as having lung cancer prior to Medicare enrollment, we believed that they would have a healthcare visit within the first three years of entry. In our cohort, beneficiaries entered the cohort on January 1 of the year following the 3-year lung cancer-free period, during which no lung cancer diagnosis occurred, and were followed until the first diagnosis of lung cancer, death, or end of the follow-up. The schematic flowchart of the study population selection was provided in Section 1 of the Supplemental Material.

Our research was approved by CMS and Institutional Review Boards of Emory University and Harvard University T.H. Chan School of Public Health.

### 2.2. Outcome assessment

The primary outcome of interest was the time to first diagnosis of primary lung cancer that began in the lungs. The first diagnoses were directly obtained from the CCW database. The CCW database used algorithms that searched from all available Medicare administrative claims (e.g., hospital inpatient and outpatient, skilled nursing facility, and carrier claims) for the first mention of a primary lung cancer diagnosis. The ICD codes used for lung cancer diagnoses by CCW included any neoplasm of the lung or bronchus, but not of the trachea. Specific ICD codes were provided in Section 2 of the Supplemental Material.

### 2.3. Exposure assessment

The exposures of interest were ZIP code-level 3-year average PM_2.5_, NO_2_, warm-season (i.e., May to October) ozone, and PR. Daily, 1-km grid, ground-level, 24-hour average PM_2.5_, 1-hour maximum NO_2_, and 8-hour maximum ozone concentrations were derived from spatiotemporal ensemble models that integrated estimates from three different machine learning algorithms, including a neural network, a gradient boosting machine, and a random forest (Di et al., 2019; Di et al., 2020; Requia et al., 2020). The ensemble models were calibrated based on several predictors including chemical transport model simulations, land-use variables, meteorological variables, satellite measurements, and air quality monitoring measurements from the Environmental Protection Agency (EPA) Air Quality System and other locations. The ensemble models yielded high performance when the estimates were assessed using 10-fold cross-validation tests against measurements recorded at EPA monitoring sites, with the resulting R^2^ for annual PM_2.5_, NO_2_, and warm-season ozone of 0.89, 0.84, and 0.86, respectively. Monthly predictions of gross beta activity, a surrogate for PR, with a spatial resolution of 32 km were obtained from a spatiotemporal ensemble model integrating estimates from nine base models (Li et al., 2021). Those models used data from the EPA’s Radiation Network, a nationwide background environmental radiation monitoring network with gross beta particle activity data collected from 129 monitors. The models also incorporated predictions such as atmospheric transport of radon and radon progeny, relative humidity, air mass sources, and long-term and seasonal trends. The ensemble model outperformed all nine base models, with an R^2^ of 0.56.

We averaged grid cell predictions across ZIP codes for each year using population weights and then assigned yearly population-weighted air pollution exposure to each subject based on their residential ZIP code. We calculated 3-year moving averages of PM_2.5_, NO_2_, warm-season ozone, and PR for each follow-up year of each subject. Changes in residential ZIP codes were considered, based on the yearly ZIP code updates from the Medicare denominator file.

### 2.4. Covariates

Individual-level covariates included sex, race (Black, White, and Other), and Medicaid eligibility (a proxy for personal socioeconomic status [SES]). These were directly obtained from the Medicare denominator file.

Neighborhood-level covariates were obtained from various sources. These include SES, behavioral risk factors, healthcare capacity, access-to-care, and meteorological and spatial trend covariates.

Neighborhood-level SES covariates included % Black population, % American Indian and Alaska Native population, % Asian population, % Two or More Races population, % Native Hawaiian and Other Pacific Islander population, % Hispanic population, % population using automobile to transport, % population receiving less than high school education, % population above 65 years of age living below the poverty line, median household income, % population living in rented houses or apartments. All of the variables involving race alone represented both Hispanics and non-Hispanics. These variables were retrieved at the ZIP code tabulation area level from the 2000 and 2010 decennial Censuses and American Community Survey 5-year estimates from 2011 to 2016 (Bureau, 2000; Bureau UC, xxxx; Bureau, 2010). Data from all other years and missing values were imputed using linear interpolation and linked to ZIP codes.

Neighborhood-level behavioral risk factors covariates included mean body mass index (BMI) and smoking rate. These variables were collected at the county level and provided by the 2001–2016 Behavioral Risk Factor Surveillance System (CDC, 2015). The data were linked to relevant ZIP codes and temporally interpolated using linear regression to fill in missing values.

Neighborhood-level healthcare capacity covariates included the number of hospitals, number of active medical doctors, and number of hospital beds regularly maintained for inpatients. These variables were collected at the county level and provided by the 2010, 2015, and 2018 American Hospital Association Annual Survey Database (Association, 2005). The data were linked to relevant ZIP codes. Data from all other years and missing values were filled using linear interpolation and extrapolation.

Neighborhood-level access-to-care covariates included distance to the nearest hospital in km, % of Medicare enrollees having at least one ambulatory visit to a primary care clinician in a year, and % of diabetic Medicare enrollees aged 65–75 having a hemoglobin A1c test in a year. Distance to the nearest hospital was calculated from the centroid of ZIP code using hospital locations across the US derived from the 2010 ESRI USA Hospitals ArcGIS database (Data, 2010). The other two neighborhood-level access-to-care covariates were collected at the hospital service area-level and provided by the 2003–2015 Dartmouth Atlas of Health Data (Cronenwett and Birkmeyer, 2000). The data were linked to relevant ZIP codes. Data from all other years and missing values were filled using linear interpolation and extrapolation.

Neighborhood-level meteorological and spatial trend covariates included normalized difference vegetation index (NDVI), population density, and population-weighted annual mean temperature in Celsius. NDVI was collected at a 5-km grid resolution from 2001 to 2016 NASA’s MODIS satellite data (Justice et al., 1998). Population density data was collected at 1-km grid resolution in 2000, 2005, 2010, 2015, and 2020 and was downloaded from NASA’s SEDAC database (Warszawski et al., 2016). The population-weighted annual mean temperature was obtained at 1-km grid resolution, aggregated from 2001 to 2016 monthly NASA Daymet and previously mentioned population density data (Thornton et al., 1840). The data were linked to relevant ZIP codes by matching centroids. Data from all other years and missing values were filled using linear interpolation.

More details on covariates data can be found elsewhere (Danesh Yazdi et al., 2021).

### 2.5. Statistical analysis

We fitted Cox proportional hazards models to estimate the association between long-term exposure to PM_2.5_, NO_2_, warm-season ozone, and PR and the incidence of lung cancer among the Medicare population. The time scale used was age in years. We fitted single- and multi-pollutant models to estimate the hazard ratios (HRs) per unit increase in the 3-year average PM_2.5_ (μg/m^3^), NO_2_ (ppb), warm-season ozone (ppb), and PR (mBq/m^3^) concentrations. To control for potential confounding, we adjusted for individual-level covariates (sex, race, and Medicaid eligibility) and neighborhood-level SES, behavioral risk factor, healthcare capacity, access-to-care, and meteorological and spatial trend covariates. We also conducted subgroup analyses in single- and multi-pollutant models restricting to person-years with pollution levels of PM_2.5_ ≤ 10 μg/m^3^, NO_2_ ≤ 30 ppb, warm-season ozone ≤ 35 ppb, and PR ≤ 8 mBq/m^3^. The threshold levels for PM_2.5_ and NO_2_ were below current ambient air pollution standards in the US. There was no standard for long-term ozone, nor any standards for PR.

We further assessed the potential effect measure modification by conducting subgroup analyses in our multi-pollutant model stratifying by individual-level characteristics sex, age, race, and Medicaid eligibility, and by neighborhood-level characteristics median household income, smoking rate, and poverty rate.

To test the robustness of our findings, we conducted a series of sensitivity analyses. We repeated the analysis using a 1- and 5-year air pollution exposure averages for the cohort with a 3-year lung cancer-free period. We also constructed another sub-cohort, with a stricter definition of a 5-year lung cancer-free period, and repeated the analysis using a 1-, 3-, and 5-year air pollution exposure average. In addition, we used stabilized inverse probability weights based on a propensity score model for air pollution to obtain causal estimates (Wei et al., 2021). Methodology details for the propensity score model were provided in Section 3 of the Supplementary Material.

All analyses were run in R software, version 3.6.1. A two-sided p-value of less than 0.05 was considered statistically significant.

### 2.6. Data disclaimer

Medicare data used in this study cannot be shared nor made publicly available due to restrictions in the Data Use Agreement with the CMS. Researchers interested in Medicare data should request data from CMS directly and complete separate Data Use Agreements. PM_2.5_, NO_2_, and ozone data are publicly available on the SEDAC websites (Di et al., 2000; Di et al., 2000; Requia et al., 2000; Wei et al., 2022). PR data are available upon request from the corresponding author. The covariate data are publicly available, with sources described in the manuscript. Data were analyzed at Emory Rollins School secure cluster environment (HPC), certified for use with confidential health records data storage and analysis. Data was safeguarded in compliance with Health Insurance Portability and Accountability Act (HIPAA).

## 3. Results

The final cohort consisted of 12.4 million fee-for-service Medicare beneficiaries who were continuously enrolled in both Medicare Part A and Part B between 2004 and 2016 and had a 3-year period of free of lung cancer diagnoses following their enrollment in Medicare (Table 1). The median follow-up was 7.7 years. We identified 166,860 lung cancer events over the study period, representing about 1.3 % of the population. The average age at entry was 71.5 years and there was a higher proportion of women (59.2 %) than men (40.8 %). Most participants were Whites (89.7 %) and never enrolled in Medicaid (89.6 %).

The 3-year average PM_2.5_ across person-years over the study period was 9.6 ± 2.5 μg/m^3^. For NO_2_, the 3-year average concentration was 18.9 ± 8.5 ppb. For warm-season ozone, the 3-year average concentration was 42.5 ± 5.4 ppb and, for PR, the 3-year average concentration was 9.8 ± 1.0 mBq/m^3^. The distributions for all pollutants were slightly skewed, with outliers presented on the right sides of the distributions (Table 2). The 3-year average levels of PM_2.5_ and PR showed a relatively strong correlation (correlation coefficient = 0.47), while other pairwise correlations were low (Section 4 of Supplementary Material).

Long-term exposure to NO_2_ was significantly associated with an increased risk of lung cancer for all model specifications (Table 3), including those restricted to low exposure concentration levels (NO_2_ ≤ 30 ppb). Long-term exposure to PM_2.5_ was significantly associated with an increased risk of lung cancer for all model specifications, except for the multi-pollutant model restricted to low exposure concentration levels (PM_2.5_ ≤ 10 μg/m^3^), where insignificant association was found. Long-term exposure to warm-season ozone was marginally associated with an increased risk of lung cancer in the low-exposure models (warm-season ozone ≤ 35 ppb), while significant negative associations were observed in models for the full cohort. Long-term exposure to PR was significantly associated with an increased risk of lung cancer for all model specifications, except for the multi-pollutant model for the full cohort, where the association was marginally significant.

When restricting exposures to low levels, effect estimates for NO_2_ were slightly larger for both single- and multi-pollutant models. For PM_2.5_, we observed weaker effect in the single-pollutant model and insignificant effect in the multi-pollutant model. For warm-season ozone, there was a shift from a negative association to positive association at low exposure levels for both single- and multi-pollutant models. For PR, effect estimates at low exposure levels were substantially larger in both single- and multi-pollutant models compared with those among the full cohort.

**Fig. 1.**
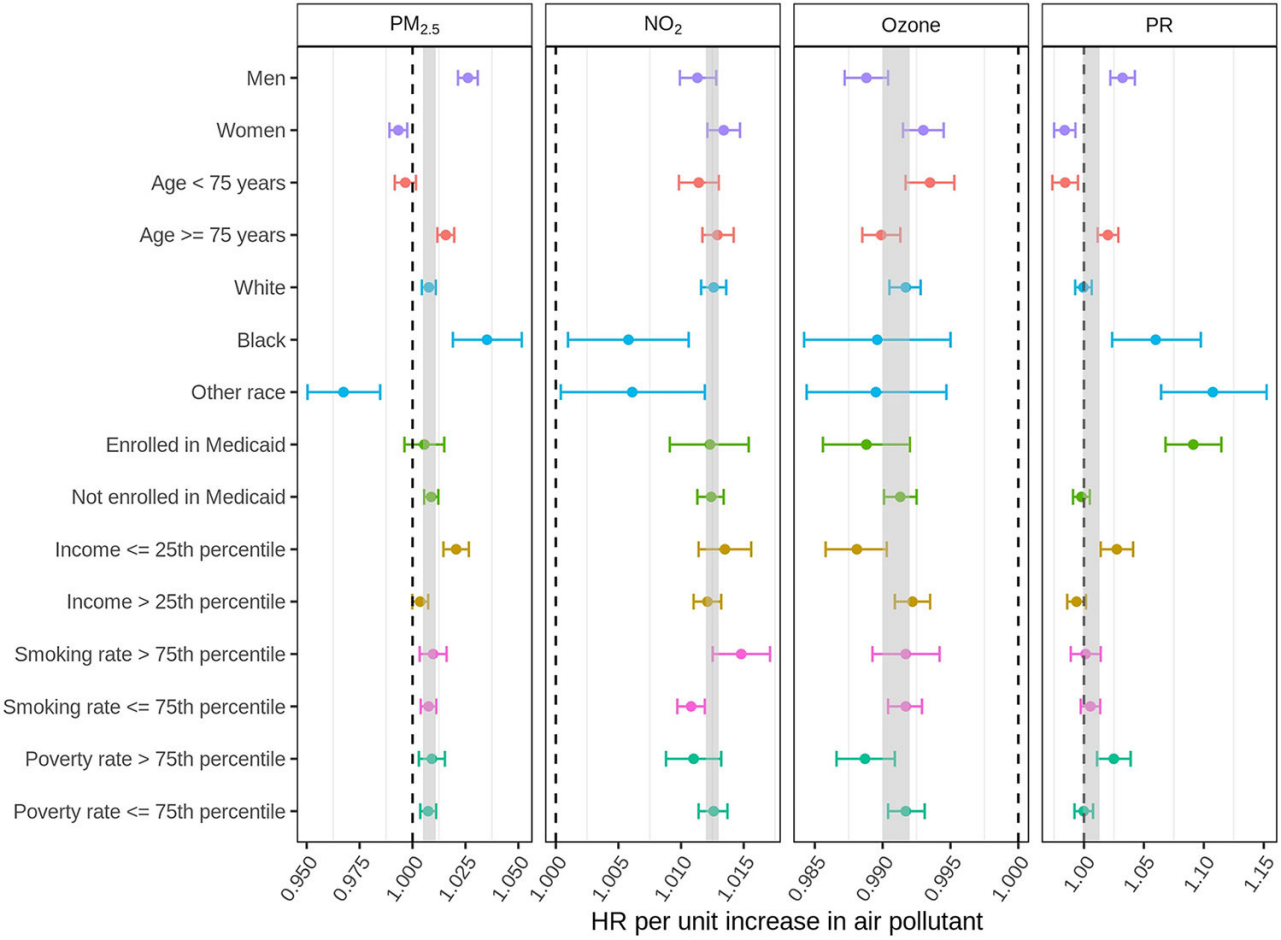
Hazard ratios of lung cancer incidence associated with one unit increase in 3-year average PM_2.5_ (μg/m^3^), NO_2_ (ppb), warm-season ozone (ppb), and PR (mBq/m^3^) exposures by subgroup in Medicare cohort from 2004 to 2016. All results were derived from the multi-pollutant models. The grey area denotes the 95% CI of the overall estimates for each pollutant in the multi-pollutant model with full cohort.

We further evaluated effect measure modification by conducting subgroup analysis stratifying on individual- and neighborhood-level characteristics in the multi-pollutant model (Fig. 1). Results stratifying on individual-level characteristics vary by pollutant. For PM_2.5_, there was a significantly higher risk of lung cancer among men, individuals older than 75 years, and Blacks. For NO_2_, Blacks and other racial groups had a lower risk of lung cancer. For warm-season ozone, we did not observe significant differences between subgroups. For PR, men, individuals older than 75 years, Blacks and other racial groups, and those who were eligible for Medicaid experienced a higher lung cancer risk. Results stratifying on neighborhood-level characteristics showed that individuals living in ZIP codes with the lowest quarter of median household income experienced higher lung cancer risk for PM_2.5_ and PR. Numeric results from subgroup analysis were provided in Section 5 of the Supplemental Material.

Our results remained robust in sensitivity analyses. In analyses using 1- and 5-year average exposures, we found similar results for NO_2_ and warm-season ozone and slight attenuation of the effect for PM_2.5_ at the 5-year average exposure window (Section 6 of Supplementary Material). The effect for PR reversed for the 1-year average exposure but increased in magnitude for the 5-year average exposure. In the sub-cohort with a lung cancer-free period of 5 years, again, we found similar results for NO_2_ and warm-season ozone, slight attenuation of the effect for PM_2.5_ for the 5-year average exposure, and reversed effect estimates for PR. In the propensity score model, the effects for all pollutants remained consistent with those from the main analysis (Section 7 of Supplementary Material).

## 4. Discussion

In this large nationwide cohort composed of the elderly population in the US, long-term exposures to PM_2.5_, NO_2_, and PR were independently associated with an increased risk of lung cancer incidence. The associations persisted at concentrations below the current national standards for PM_2.5_ and NO_2_, as well as at low concentrations for PR. For warm-season ozone, we found positive associations with increased risk of lung cancer when restricting to low exposure levels. Men, older individuals, Blacks, and residents of low-income neighborhoods experienced higher incidence of lung cancer under the effects of PM_2.5_ and PR.

Existing literature investigating the effects of air pollution on lung cancer mostly focused on lung cancer mortality, possibly due to better data availability. Lung cancer has a high mortality rate, with an estimated 5-year survival of 18.6 % (Ganti et al., 2021). However, investigating incidence offers unique insights, as it allows us to understand the role that air pollution in the development of lung cancer, as opposed to progression. Such insights are informative for preventive strategies, which might be of more importance in reducing cancer burden.

Our results for PM_2.5_ and NO_2_ in the single-pollutant model were consistent with the results of two *meta*-analyses studies conducted by Hamra et al. (Hamra Ghassan et al., 2014) and Hamra et al. (Hamra Ghassan et al., 2015), who investigated lung cancer incidence and mortality jointly. Both *meta*-analyses included large cohort studies such as the European Study of Cohorts for Air Pollution Effects (ESCAPE), American Cancer Society Cancer Prevention Study, NHS, and the Canadian National Enhanced Cancer Surveillance System Case-Control study. The pooled relative risk for lung cancer associated with 10 μg/m^3^ increase in PM_2.5_ and NO_2_ was 1.09 (95 % CI: 1.04, 1.14) and 1.04 (95 % CI: 1.01 and 1.08), respectively. Both estimates were lower in magnitude when compared to our results, where we found lung cancer HRs of 1.16 (95 % CI: 1.13, 1.20) associated with 10 μg/m^3^ increase in PM_2.5_ and of 1.07 (95 % CI: 1.06, 1.07) associated with 10 μg/m^3^ increase in NO_2_, based on a conversion factor of 1 ppb = 1.88 μg/m^3^ and the single pollutant models. Our study used air pollution estimates derived from a large ensemble that had excellent prediction accuracy. Since exposure error likely attenuated associations (Wei et al., 2022), the more precise estimates from our model could lead to discovery of stronger associations that are larger in magnitude compared to studies incorporated in the two *meta*-analyses that utilized estimates based to the nearest air monitor site, land-use regression models, spatiotemporal models, and air dispersion models. Moreover, our study was conducted among the elderly population, with an average age of 71.5 years. Although we controlled for age by using it as the time scale in the Cox proportional hazards model, there could be some effect modification by age which may explain the slightly higher estimates in our population. The IARC has identified air pollution, and PM_2.5_ specifically, as group 1 carcinogen for lung cancer. As a complex mixture, PM_2.5_ includes various metals and hydrocarbons associated with DNA damage and repair changes, inflammation, altered immune and oxidative stress responses, and epigenetic alterations such as DNA methylation (Loomis et al., 2013). For NO_2_, consistent evidence has associated it with the development of lung cancer (Hamra Ghassan et al., 2015). Although NO_2_ itself is not an established carcinogen, it is a surrogate for traffic-related pollution, which includes a variety of diverse carcinogens such as volatile organic compounds (VOCs) and polycyclic aromatic hydrocarbons (Bai et al., 2020). The association of NO_2_ in the present study provided further support for the carcinogenicity of previously identified traffic-related pollutants. It may also reflect unidentified carcinogenic properties of NO_2_.

Few studies have investigated the effects of warm-season ozone and lung cancer and most of these report null results. Kazemiparkouhi et al. (Kazemiparkouhi et al., 2020) investigated lung cancer mortality among the Medicare population (~22.2 million people) from 2000 to 2008 using log-linear regression models and found a lung cancer mortality relative risk (RR) of 1.016 (95 % CI: 1.011–1.020) per 10 ppb increase in warm-season ozone. Other large prospective cohort studies in Canada (~4.9 million people) (Bai et al., 2020) and Europe (~0.3 million people) (Hvidtfeldt et al., 2021) and a population-based case-control study in Canada (~0.6 million people) found null results. In our study, warm-season ozone was associated with increased lung cancer incidence only in low-exposure analyses (warm-season ozone ≤ 35 ppb), although the effects were marginally significant (Table 3). One possible explanation for the negative associations with lung cancer for warm-season ozone in the full cohort comes from the spatial distribution of warm-season ozone across the US. High warm-season ozone concentrations in our study were found mostly in the Rocky Mountains and Central Valley of California region of the US (Section 7 of Supplementary Material). These were areas with likely more non-anthropogenic sources of ozone, especially ground-level ozone vertically transported from the stratosphere (also called stratospheric ozone intrusions) which was more likely to occur in high-elevation areas such as Rocky Mountains (Simon et al., 2015). The terrain based on mountains and valleys in the region could also possibly lead to an accumulation of ozone transported from neighboring regions by wind. It is possible that there was a difference in health effects associated with anthropogenic and non-anthropogenic ozone, with the former being more harmful to human health. Anthropogenic ozone was formed from photochemical reactions with other pollutants such as traffic-originated NO_2_ and VOCs. As such, ozone concentrations could generally serve as a proxy for other anthropogenic photochemical pollutants and VOCs harmful to human health in low-level areas (Bai et al., 2020). Supporting this hypothesis, we found a higher correlation between ozone and NO_2_ in the low-level regions where ozone levels remained consistently below 35 ppb (Section 8 of Supplementary Material). However, this was not the case for naturally occurring ozone in the Rocky Mountains and Central Valley of California, which might explain why we saw negative associations for ozone in regions where ozone levels not remained consistently below 35 ppb (Section 9 of Supplementary Material). The previously mentioned Medicare lung cancer mortality study (Kazemiparkouhi et al., 2020) that found positive associations for warm-season ozone assigned warm-season ozone exposure to each study subject based on warm-season ozone concentrations measured at the air quality monitor closest to the centroid of the ZIP code of residence. In contrast, our study assigned exposure to subjects based on predicted warm-season ozone exposure at each ZIP code based on a large-ensemble model, which could also explain some differences. In the US, air quality monitors were not evenly distributed across the country, with a lack of monitors in sparsely-populated regions, exactly where we saw high warm-season ozone exposure levels based on our large-ensemble model.

To our knowledge, no study to date has evaluated the effect of the radioactive characteristics of particles on lung cancer. Although studies have suggested an increased risk of lung cancer following radiation exposures from radiotherapy, occupational settings, or atomic bomb (Kyoji et al., 2010; Vrijheid et al., 2007; Vacquier et al., 2008), possibly through DNA damage, there is little evidence concerning environmental radioactivity. PR was emitted from radioactive radon progeny that attaches to particles (Li et al., 2021). Since we had adjusted for PM_2.5_, the identified effect estimate for PR suggested an independent effect on lung cancer development. In the low-level analysis, the substantially large effect estimates revealed a non-linear concentration–response relationship. These findings underscored the need for further research to explore the underlying biological mechanisms that may explain these observed effects. Additionally, the results called for the development of appropriate regulatory standards that took these complex relationships into account, to ensure that public health was adequately protected.

In our study population, PM_2.5_ and NO_2_ exposure levels were below the EPA annual standard of 12 μg/m^3^ and 53 ppb (National Ambient Air Quality Standards Table, 2016), respectively (Table 2). The EPA does not have a standard for warm-season ozone levels but, in our study, mean warm-season ozone levels were well below the EPA standard for daily maximum 8-hour average ozone of 70 ppb. We found increased lung cancer risk in the cohort restricted to low exposure levels (PM_2.5_ ≤ 10 μg/m^3^, NO_2_ ≤ 30 ppb, and warm-season ozone ≤35 ppb) associated with all three pollutants in both single- and multi-pollutant models, suggesting that even at concentrations below current national standards, air pollution was associated with an increased risk of lung cancer.

Comparing the results from single- and multi-pollutant models, we observed some confounding effects between air pollutants. Effect estimates for NO_2_ were consistent in all model specifications, whereas the effect estimates for PM_2.5_ and PR were attenuated and were stronger for warm-season ozone (in the negative direction), comparing the multi-pollutant model to single-pollutant models (Table 3). The robustness of NO_2_ results suggested that traffic-related air pollution might be a strong risk factor for lung cancer, independently of other sources of air pollution. The attenuated effects of PM_2.5_ and PR in the multi-pollutant model might because of their common sources. For instance, traffic emissions contributed to both PM_2.5_ and a significant portion of NO_2_. In our results, the reduction in the PM_2.5_ effect size when controlling for NO_2_ was consistent with this relationship, indicating that in the single pollutant model, PM_2.5_ likely captured effects also attributable to other traffic-related pollutants. The stronger correlation between PM_2.5_ and NO_2_ in regions with higher population density supports that these pollutants are more closely linked in urban areas (Section 10 of Supplementary Material). The effect of PM_2.5_ remained significant when controlling for NO_2_, indicating the relevance of non-traffic sources. While the complexity of these interactions highlighted the importance of considering multiple pollutants simultaneously, very few studies had done so, and those that had, found mixed results. In a pooled analysis of seven European cohorts (Hvidtfeldt et al., 2021), no confounding effect by NO_2_ on PM_2.5_ was observed. In two studies investigating the effects of NO_2_ (Eum et al., 2022) and warm-season ozone (Kazemiparkouhi et al., 2020) on lung cancer mortality among the Medicare cohort, the estimate for NO_2_ remained robust after the adjustment for PM_2.5_, and the estimate for warm-season ozone remained robust after adjustment for NO_2_ and PM_2.5_. All these studies found positive associations between air pollutants investigated and lung cancer risk.

In our subgroup analysis, we found an increased risk of lung cancer attributable to PM_2.5_ and PR for men, Blacks, those older than 75, and those living in neighborhoods with lower median household income. We also found a decreased risk of lung cancer attributable to NO_2_ for Blacks and other racial groups. Some other studies have investigated the risk of lung cancer mortality or incidence by subgroups other than smoking status. Bai et al. (Bai et al., 2020) conducted a large-cohort study in Ontario Population Health and Environment Cohort and found that people living in lower income areas had high incidence of lung cancer associated with PM_2.5_, consistent with our results. Eum et al. (Eum et al., 2022) found a higher NO_2_-associated lung cancer mortality for Blacks compared to Whites among the Medicare cohort, in contrast to our results. Unmeasured confounders might explain this difference. Compared to previous studies, we adjusted for more neighborhood-level covariates, including SES, access-to-care, and behavioral risk factors, which were closely tied to race. In terms of effect modification by smoking, a previous meta-analysis study (Hamra Ghassan et al., 2014) found the highest lung cancer risk associated with PM_2.5_ for former smokers, followed by never-smokers, and current smokers. In our study, we found no significant difference in lung cancer risk associated with PM_2.5_ between subgroups residing in neighborhoods with higher or lower smoking rates.

We also evaluated different lag periods for air pollution exposures. We hypothesized that if a shorter lag between air pollution exposure and lung cancer had a larger effect estimate, assuming a causal relationship, it would imply that air pollution has a promoter role in accelerating the development of lung cancer. On the other hand, if a longer lag had a larger effect estimate, air pollution probably had a more relevant role in initiating the onset of lung cancer. In our study, estimates for NO_2_ and warm-season ozone remained consisted in analysis using 1-, 3-, and 5-year average air pollution exposure (Section 6 of Supplementary Material). For PM_2.5_, we see a slight reduction in the effect at a 5-year average exposure window compared to 1- and 3-year average, suggesting a stronger role in accelerating the progression of lung cancer. On the contrast, for PR, the increase in the effect size with exposure time frame suggests a stronger role in initiating the onset of lung cancer.

Our results remained robust in sensitivity analyses. When repeating the analysis in a sub-cohort of a 5-year lung cancer-free period, results remained consistent at 3-year average air pollution exposure, except for PR (Section 6 of Supplementary Material). In the causal framework using inverse probability weights based on propensity score models, results remained robust (Section 7 of Supplementary Material). Few existing studies have employed causal modeling to investigate the effects of air pollution exposures on lung cancer. In the present study, the consistency between associational and causal estimates enhanced the robustness of our findings, identified the causal pathway, and thereby offered direct policy implications.

Our study has several strengths. Firstly, we were able to obtain precise air pollution exposure estimates from high-resolution large ensemble models that showed excellent predictive accuracy. These models provided better estimates for ZIP-code level air pollution than estimates based on the distance to the nearest air quality monitor or land-use regression and allowed us to estimate exposures across the contiguous US. Secondly, the large sample size gave us ample power to detect small differences, which is crucial when studying lung cancer that had a relatively low incidence. Thirdly, our strict requirement for a 3-year lung cancer-free period and inclusion criteria to include only Medicare beneficiaries enrolled in the fee-for-service program and both Part A (hospital insurance) and Part B (medical insurance) program allowed us to correctly identify newly diagnosed lung cancer cases. The Medicare CCW dataset included claims from a wide range of sources such as doctor visits, hospitalization records, outpatient visits, and skilled nursing facilities, which allowed us to ascertain lung cancer incidence with some assurance when compared to studies looking at limited administrative records. Lastly, we were able to control for a comprehensive set of covariates, especially neighborhood-level covariates. Compared to the results from the main analysis, the model that did not adjust for neighborhood-level covariates suggested that these factors have a substantial impact on the effect estimates (Section 11 of Supplementary Material).

Some limitations in our study should be noted. First, our results were limited by the lack of individual-level risk factors, such as smoking habit, occupational exposures such as to asbestos and other carcinogens, and family history. Consequently, we were unable to evaluate the relationship between air pollution and lung cancer risk between subgroups by these factors. As for residual confounding, it was unlikely that our results were biased due to these unmeasured factors, as they were not correlated with ambient air pollution predictions. Besides, our study was limited by its generalizability. Our study was restricted to a selection of Medicare beneficiaries who were enrolled in the fee-for-service program and both Part A and Part B programs. Further work is needed to determine whether the association found is generalizable to the entire US elderly population or in other countries.

## 5. Conclusion

This was the first nationwide cohort study investigating the simultaneous effects of PM_2.5_, NO_2_, warm-season ozone, and PR on lung cancer incidence in the US. Our study provided robust evidence that long-term exposures to PM_2.5_, NO_2_, and PR were independently associated with an increased risk of lung cancer incidence. At low exposure levels, all examined pollutants were associated with increased incidence of lung cancer. Importantly, the levels for PM_2.5_ and NO_2_ were below the national air quality standards, indicating that existing standards may not adequately safeguard public health. Men, older individuals, Blacks, and residents of low-income neighborhoods experienced larger effects of PM_2.5_ and PR. This study adds to the growing body of evidence that calls for more stringent and urgent air quality regulations.

## Supplementary Material

1

## Figures and Tables

**Table 1:** Descriptive characteristics of Medicare beneficiaries who were continuously enrolled in both Medicare Part A and Part B between 2004 and 2016 and had a 3-year period of free of lung cancer diagnoses following their enrollment in Medicare.

Variable	Value
**Cohort description**	
**Total population**	12,429,951 (100 %)
**Total person-years**	95,280,525
**Number of events**	166,860 (1.3 %)
**Median follow-up** (years)	7.7
**Individual characteristics**	
**Age at study entry** (years)	71.5 ± 5.0
**Sex**	
Men	5,070,128 (40.8 %)
Woman	7,359,823 (59.2 %)
**Race**	
White	11,153,635 (89.7 %)
Black	690,780 (5.6 %)
Other	585,536 (4.7 %)
**Ever enrolled in Medicaid**	
Yes	1,287,642 (10.4 %)
No	11,142,309 (89.6 %)

**Table 2:** Distributions of 3-year average air pollution exposures across person-years in Medicare cohort from 2004 to 2016.

Pollutant	Mean ± SD	Minimum	25th Percentile	Median	75th Percentile	Maximum
PM_2.5_ (μg/m^3^)	9.6 ± 2.5	0.8	8.0	9.5	11.2	24.5
NO_2_ (ppb)	18.9 ± 8.5	0.6	12.5	17.1	24.0	79.2
Warm-season ozone (ppb)	42.5 ± 5.4	20.7	39.8	42.6	45.4	79.4
PR (mBq/m^3^)	9.8 ± 1.0	5.9	9.2	10.0	10.5	14.4

**Table 3:** Hazard ratios of lung cancer per unit increase in 3-year average PM_2.5_ (μg/m^3^), NO_2_ (ppb), warm-season ozone (ppb), and PR (mBq/m^3^) exposures in single- and multi-pollutant models in Medicare cohort from 2004 to 2016.

Model	PM_2.5_	NO_2_	Warm- season ozone	PR
**Single-Pollutant**				
Full cohort ^a^	1.015 (1.012, 1.018)	1.012 (1.011, 1.012)	0.997 (0.996, 0.998)	1.016 (1.010, 1.022)
Low-exposure cohort ^b^	1.007 (1.001, 1.012)	1.014 (1.013, 1.016)	1.008 (0.999, 1.016)	1.134 (1.059, 1.214)
**Multi-pollutant** ^a^				
Full cohort ^a^	1.008 (1.005, 1.011)	1.013 (1.012, 1.013)	0.991 (0.990, 0.992)	1.005 (0.999, 1.012)
Low-exposure cohort ^b^	1.004 (0.998, 1.010)	1.016 (1.015, 1.017)	1.005 (0.996, 1.013)	1.125 (1.046, 1.210)

a Analysis conducted in full cohort (PYs = 95,280,525, lung cancer events = 166,860).

b Analysis restricted to person-years with exposure levels ≤ 10 μg/m^3^ for PM_2.5_ (PYs = 55,771,344, lung cancer events = 94,429), ≤ 30 ppb for NO_2_ (PYs = 84,120,835, lung cancer events = 145,449), ≤ 35 ppb for warm-season ozone (PYs = 7,584,079, lung cancer events = 13,344), and ≤ 8 mBq/m^3^ for PR (PYs = 2,951,296, lung cancer events = 4,620). All models were adjusted for sex, race (Black/White/Other), Medicaid eligibility (yes/no), temperature, population density, % Black population, % American Indian and Alaska Native population, % Asian population, % Two or More Races population, % Native Hawaiian and Other Pacific Islander population, % Hispanic population, % population using automobile to transport, % population receiving less than high school education, % population above 65 years of age living below the poverty line, median household income, % population living in rented houses or apartments, distance to the nearest hospital, % of Medicare enrollees having at least one ambulatory visit to a primary care clinician in a year, % of diabetic Medicare enrollees aged 65–75 having hemoglobin A1c test in a year, number of hospitals, number of medical doctors, number of hospital beds, NDVI, BMI, and smoking rate. The multi-pollutant models were additionally adjusted for other pollutants.

## Data Availability

Please refer to “Data Disclaimer” section in the revised manuscript.
