# Supplementary material for: Long-term association of air pollution and incidence of lung cancer among older Americans: A national study in the Medicare cohort
Source: Environ Int. Author manuscript; Available in PMC 2023 Dec 1. (PMC10691920; doi:10.1016/j.envint.2023.108266)
Supplement: 1 [file NIHMS1945847-supplement-1.docx]

**Supplementary Material**

**Section 1. Schematic flowchart of the study population selection**


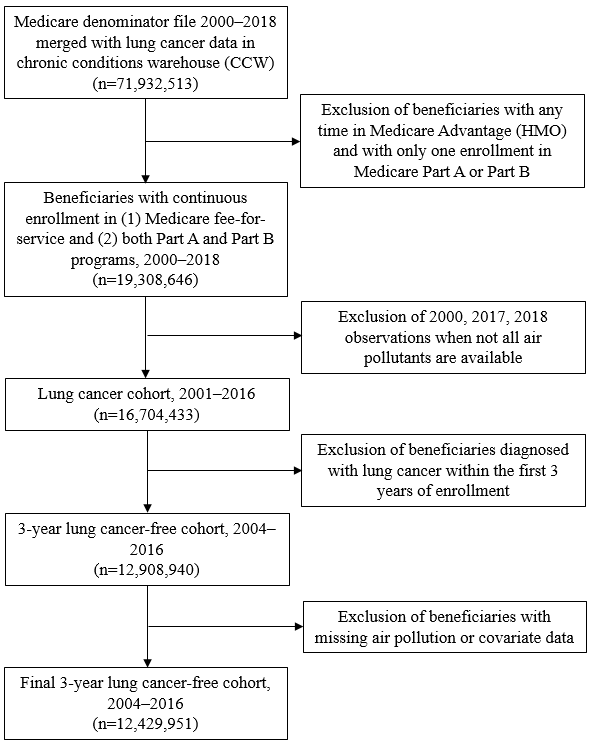


**Section 2. ICD-codes for lung cancer used in Medicare Chronic Conditions Warehouse (CCW) database ^a^**

|  | **Lung Cancer** |
| --- | --- |
| **ICD-9** | 162.2, 162.3, 162.4, 162.5, 162.8, 162.9, 231.2, V10.11 (any DX on the claim) |
| **ICD-10** | C34.00, C34.01, C34.02, C34.10, C34.11, C34.12, C34.2, C34.30, C34.31, C34.32, C34.80, C34.81, C34.82, C34.90, C34.91, C34.92, D02.20, D02.21, D02.22, Z85.110, Z85.118 (any DX on the claim) |

^a^ On October 1, 2015, Medicare switched from using the *Ninth Revision of the International Classification of Diseases (ICD-9)* to *Tenth Revision (ICD-10)*. More details can be found elsewhere ^1^.

**Section 3. Methodology details and distribution of propensity scores estimated in the sensitivity analysis**

For each pollutant, the propensity score analysis consisted of a design stage and an analysis stage.

In the design stage, we created a “pseudo-population” by weighting the observed population by the inverse probability of being assigned to the observed exposure, given all measured confounders. The inverse probability weight for person-year $i$ was defined by ${sw}_{i}=\frac{\bar{T}}{\hat{R}_{i}}$, where $\bar{T}$ was the average exposure level across all person-years, and $\hat{R}_{i}$ was the estimated generalized propensity score (GPS) obtained from a linear regression of the observed exposure ($T_{i}$) against all measured confounders ($C_{i}$). Specifically, the GPS for person-year $i$ was estimated by $\hat{R}_{i}=\frac{1}{\sqrt{2\pi\hat{\sigma}^{2}}}exp\left( -\frac{1}{2\hat{\sigma}^{2}}\left( T_{i}-\hat{T}_{i} \right)^{2} \right)$, where $\hat{T}_{i}$ is the predicted exposure level based on the fitted linear model. To account for outliers, ${sw}_{i}$ was truncated so that values above 97.5th percentile were given the value of 97.5th percentile, and values below 2.5th were given the value of 2.5th percentile.

In the analysis stage, we fitted a Cox proportional hazards model of the time to lung cancer diagnosis against the exposure, weighted by ${sw}_{i}$.

Figure below shows the distributions of ${sw}_{i}$ for each pollutant across all ZIP code-year combinations.


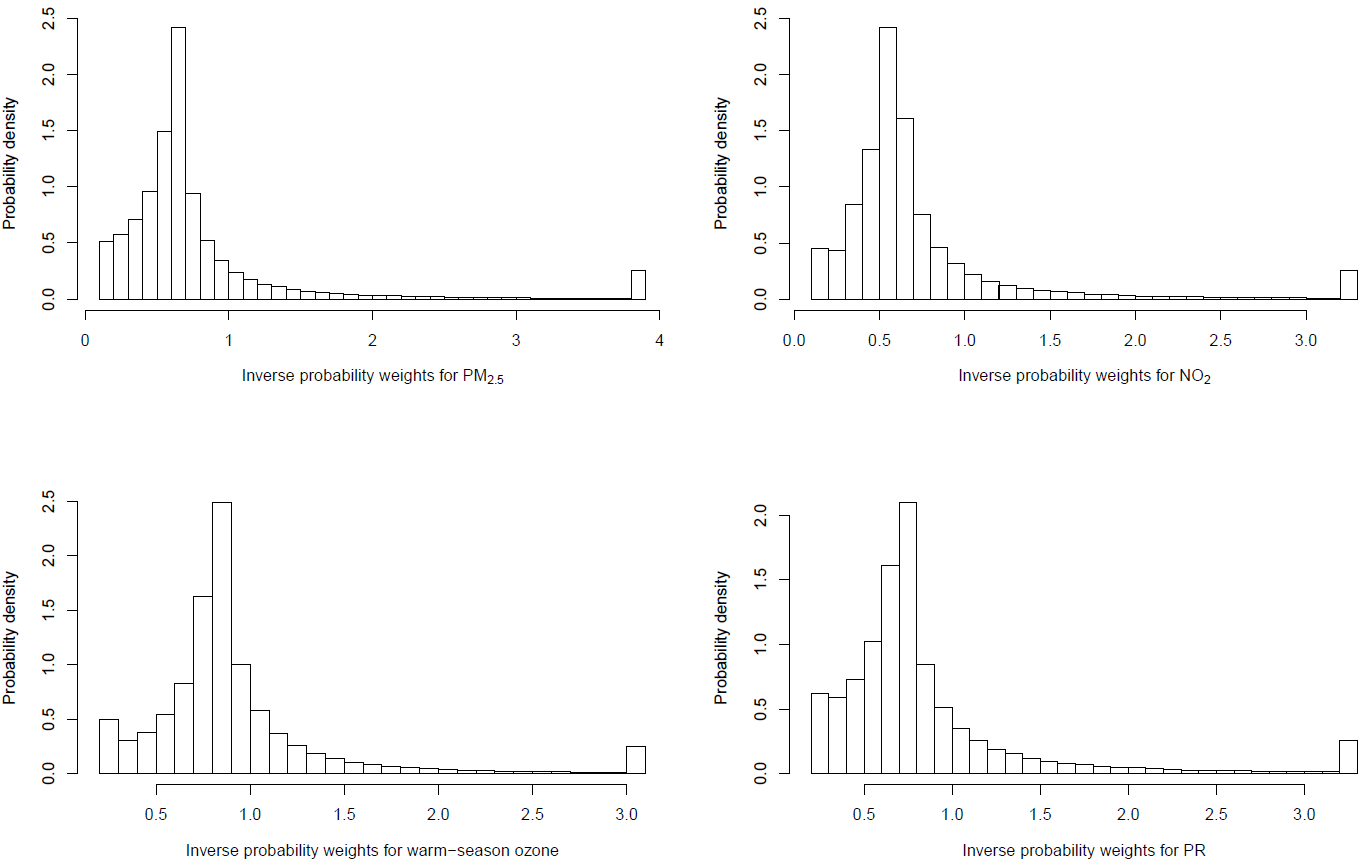
**Figure S1. Distributions of inverse probability weights for each air pollutant across all person-years of follow-up.**

**Section 4. Pairwise correlation coefficients among 3-year average exposures to PM_2.5_ (μg/m^3^), NO_2_ (ppb), warm-season ozone (ppb), and PR (mBq/m^3^)**

|  | **PM_2.5_** | **NO_2_** | **Warm-season ozone** | **PR** |
| --- | --- | --- | --- | --- |
| **PM_2.5_** | 1 | 0.38 | 0.30 | 0.47 |
| **NO_2_** |  | 1 | 0.24 | 0.16 |
| **Warm-season ozone** |  |  | 1 | 0.36 |
| **PR** |  |  |  | 1 |

**Section 5. Hazard ratios of lung cancer per unit increase in 3-year average PM_2.5_ (μg/m^3^), NO_2_ (ppb), warm-season ozone (ppb), and PR (mBq/m^3^) exposures in multi-pollutant models for subgroups in Medicare cohort from 2004–2016**

| **Category** | **Subgroup** | **Person-years** | **Lung cancer cases** | **PM_2.5_** | | **NO_2_** | **Warm-season ozone** | **PR** |
| --- | --- | --- | --- | --- | --- | --- | --- | --- |
| **Individual characteristics** | | | | | | | | |
| Age | < 75 years | 42,419,412 | 62,767 | 0.997 (0.992, 1.002) | | 1.011 (1.010, 1.013) | 0.994 (0.992, 0.995) | 0.984 (0.974, 0.995) |
|  | ≥ 75 years | 52,861,113 | 104,093 | 1.016 (1.012, 1.020) | | 1.013 (1.012, 1.014) | 0.990 (0.989, 0.991) | 1.020 (1.011, 1.029) |
| Sex | Men | 37,139,995 | 76,215 | 1.026 (1.022, 1.031) | | 1.011 (1.010, 1.013) | 0.989 (0.987, 0.990) | 1.032 (1.022, 1.042) |
|  | Women | 58,140,530 | 90,645 | 0.993 (0.989, 0.998) | | 1.013 (1.012, 1.015) | 0.993 (0.992, 0.995) | 0.984 (0.975, 0.993) |
| Race | White | 86,494,733 | 1,531,90 | 1.008 (1.004, 1.011) | | 1.013 (1.012, 1.014) | 0.992 (0.991, 0.993) | 1.000 (0.993, 1.007) |
|  | Black | 5,057,014 | 9,005 | 1.035 (1.019, 1.052) | | 1.006 (1.001, 1.011) | 0.990 (0.984, 0.995) | 1.060 (1.024, 1.098) |
|  | Other | 3,728,778 | 4,665 | 0.967 (0.950, 0.985) | | 1.006 (1.000, 1.012) | 0.990 (0.984, 0.995) | 1.108 (1.064, 1.152) |
| Medicaid eligibility | Eligible | 7,162,641 | 17,981 | 1.006 (0.996, 1.015) | | 1.012 (1.009, 1.015) | 0.989 (0.986, 0.992) | 1.091 (1.068, 1.115) |
|  | Non-eligible | 8,8117,884 | 148,879 | 1.009 (1.006, 1.012) | | 1.012 (1.011, 1.013) | 0.991 (0.990, 0.993) | 0.998 (0.991, 1.005) |
| **Neighborhood characteristics** | | | | |  | | | |
| Median household income | Lowest quarter | 23,820,164 | 44,763 | 1.021 (1.015, 1.027) | | 1.014 (1.011, 1.016) | 0.988 (0.986, 0.990) | 1.027 (1.014, 1.041) |
|  | Rest | 71,460,361 | 122,097 | 1.004 (1.000, 1.007) | | 1.012 (1.011, 1.013) | 0.992 (0.991, 0.994) | 0.994 (0.986, 1.002) |
| Poverty rate | Highest quarter | 23,819,578 | 42,633 | 1.009 (1.003, 1.015) | | 1.011 (1.009, 1.013) | 0.989 (0.987, 0.991) | 1.025 (1.011, 1.039) |
|  | Rest | 71,460,947 | 124,227 | 1.007 (1.004, 1.011) | | 1.013 (1.011, 1.014) | 0.992 (0.990, 0.993) | 1.000 (0.992, 1.008) |
| Smoking Rate | Highest quarter | 23,764,877 | 44,399 | 1.010 (1.003, 1.016) | | 1.015 (1.013, 1.017) | 0.992 (0.989, 0.994) | 1.001 (0.989, 1.014) |
|  | Rest | 71,515,648 | 122,464 | 1.008 (1.004, 1.011) | | 1.011 (1.010, 1.012) | 0.992 (0.990, 0.993) | 1.005 (0.997, 1.014) |

All models were adjusted for sex, race (Black/White/Other), Medicaid eligibility (yes/no), temperature, population density, % Black population, % American Indian and Alaska Native population, % Asian population, % Two or More Races population, % Native Hawaiian and Other Pacific Islander population, % Hispanic population, % population using automobile to transport, % population receiving less than high school education, % population above 65 years of age living below the poverty line, median household income, % population living in rented houses or apartments, distance to the nearest hospital, % of Medicare enrollees having at least one ambulatory visit to a primary care clinician in a year, % of diabetic Medicare enrollees aged 65–75 having hemoglobin A1c test in a year, number of hospitals, number of medical doctors, number of hospital beds, NDVI, BMI, and smoking rate. The models were also adjusted for other pollutants.

For the variable constituting the subgroup analyzed (i.e. age, sex, race, Medicaid eligibility, median household income, poverty rate, or smoking rate), the adjustment was made through stratification rather than adding the variable as covariate in the Cox proportional hazards model.

**Section 6. Hazard ratios of lung cancer per unit increase in 1-year, 3-year, and 5-year average PM_2.5_ (μg/m^3^), NO_2_ (ppb), warm-season ozone (ppb), and PR (mBq/m^3^) exposures in multi-pollutant models for 3-year and 5-year lung cancer-free cohort**

| **Model** | **Person-years** | **Lung cancer cases** | **PM_2.5_** | **NO_2_** | **Warm-season ozone** | **PR** |
| --- | --- | --- | --- | --- | --- | --- |
| **3-year lung cancer-free cohort (2004 – 2016)** | | | | | | |
| 1-year average | 95,331,915 | 166,930 | 1.010 (1.007, 1.013) | 1.011 (1.010, 1.012) | 0.993 (0.992, 0.995) | 0.995 (0.989, 1.002) |
| 3-year average ^a^ | 95,280,525 | 166,860 | 1.008 (1.005, 1.011) | 1.013 (1.012, 1.013) | 0.991 (0.990, 0.992) | 1.005 (0.999, 1.012) |
| 5-year average | 82,832,055 | 146,318 | 1.003 (1.000, 1.006) | 1.013 (1.012, 1.014) | 0.991 (0.990, 0.993) | 1.008 (1.000, 1.015) |
| **5-year lung cancer-free cohort (2006 – 2016)** | | | | | | |
| 1-year average | 71,761,666 | 127,476 | 1.007 (1.003, 1.010) | 1.011 (1.010, 1.012) | 0.995 (0.994, 0.996) | 0.976 (0.968, 0.983) |
| 3-year average | 71,761,666 | 127,476 | 1.007 (1.004, 1.011) | 1.013 (1.011, 1.014) | 0.992 (0.991, 0.994) | 0.983 (0.976, 0.991) |
| 5-year average | 71,761,666 | 127,476 | 1.002 (0.998, 1.005) | 1.013 (1.012, 1.014) | 0.992 (0.990, 0.993) | 0.999 (0.991, 1.007) |

­^a^ Main analysis model specification

All models were adjusted for sex, race (Black/White/Other), Medicaid eligibility (yes/no), temperature, population density, % Black population, % American Indian and Alaska Native population, % Asian population, % Two or More Races population, % Native Hawaiian and Other Pacific Islander population, % Hispanic population, % population using automobile to transport, % population receiving less than high school education, % population above 65 years of age living below the poverty line, median household income, % population living in rented houses or apartments, distance to the nearest hospital, % of Medicare enrollees having at least one ambulatory visit to a primary care clinician in a year, % of diabetic Medicare enrollees aged 65-75 having hemoglobin A1c test in a year, number of hospitals, number of medical doctors, number of hospital beds, NDVI, BMI, and smoking rate. The models were also adjusted for other pollutants.

**Section 7. Hazard ratios of lung cancer per unit increase in 3-year average PM_2.5_ (μg/m^3^), NO_2_ (ppb), warm-season ozone (ppb), and PR (mBq/m^3^) exposures in multi-pollutant model with and without using stabilized inverse probability weights**

| **Model** | **PM_2.5_** | **NO_2_** | **Warm-season ozone** | **PR** |
| --- | --- | --- | --- | --- |
| Main analysis (without IPW) ^a^ | 1.008 (1.005, 1.011) | 1.013 (1.012, 1.013) | 0.991 (0.990, 0.992) | 1.005 (0.999, 1.012) |
| IPW for PS model ^b^ | 1.016 (1.013, 1.019) | 1.006 (1.005, 1.007) | 0.995 (0.994, 0.996) | 1.034 (1.027, 1.042) |

­^a^ Main analysis model specification

­ ^b^ Model using propensity score model-based stabilized inverse probability weights to adjust for covariates

All models were adjusted for sex, race (Black/White/Other), Medicaid eligibility (yes/no), temperature, population density, % Black population, % American Indian and Alaska Native population, % Asian population, % Two or More Races population, % Native Hawaiian and Other Pacific Islander population, % Hispanic population, % population using automobile to transport, % population receiving less than high school education, % population above 65 years of age living below the poverty line, median household income, % population living in rented houses or apartments, distance to the nearest hospital, % of Medicare enrollees having at least one ambulatory visit to a primary care clinician in a year, % of diabetic Medicare enrollees aged 65–75 having hemoglobin A1c test in a year, number of hospitals, number of medical doctors, number of hospital beds, NDVI, BMI, and smoking rate. The models were also adjusted for other pollutants.

**Section 7. Air pollution levels at ZIP codes in the contiguous US from 2001–2016**


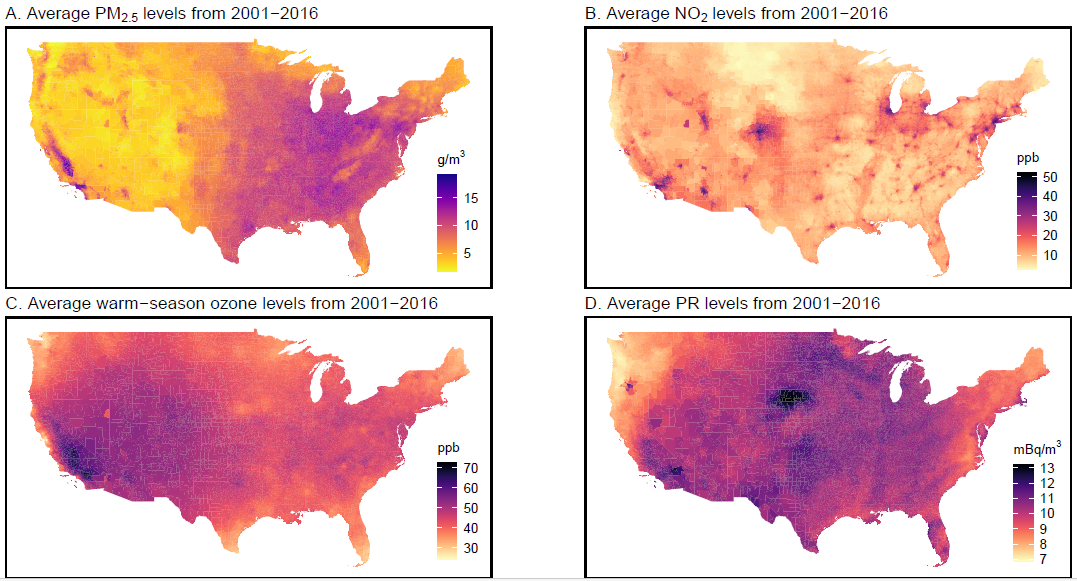


**Section 8. Pairwise correlation coefficients among 3-year average exposures to PM_2.5_ (μg/m^3^), NO_2_ (ppb), warm-season ozone (ppb), and PR (mBq/m^3^) in ZIP codes with warm-season ozone remained consistently below 35 ppb from 2001–2016 or not**

| **Warm-season ozone remained consistently below 35 ppb from 2001–2016** | | | | |
| --- | --- | --- | --- | --- |
|  | **PM_2.5_** | **NO_2_** | **Warm-season ozone** | **PR** |
| **PM_2.5_** | 1 | 0.46 | 0.48 | 0.57 |
| **NO_2_** |  | 1 | 0.16 | 0.15 |
| **Warm-season ozone** |  |  | 1 | 0.34 |
| **PR** |  |  |  | 1 |
|  | | | | |
| **Warm-season ozone not remained consistently below 35 ppb from 2001–2016** | | | | |
|  | **PM_2.5_** | **NO_2_** | **Warm-season ozone** | **PR** |
| **PM_2.5_** | 1 | 0.25 | 0.02 | 0.28 |
| **NO_2_** |  | 1 | 0.36 | 0.17 |
| **Warm-season ozone** |  |  | 1 | 0.36 |
| **PR** |  |  |  | 1 |

**Section 9. Hazard ratios of lung cancer per unit increase in 3-year average PM_2.5_ (μg/m^3^), NO_2_ (ppb), warm-season ozone (ppb), and PR (mBq/m^3^) exposures in ZIP codes with warm-season ozone remained consistently below 35 ppb from 2001–2016 or not**

| **Model** | **PM_2.5_** | **NO_2_** | **Warm-season ozone** | **PR** |
| --- | --- | --- | --- | --- |
| Warm-season ozone remained consistently  below 35 ppb from 2001–2016 | 0.995 (0.976, 1.015) | 1.008 (1.002, 1.013) | 1.014 (1.006, 1.023) | 1.030 (1.004, 1.056) |
| Warm-season ozone not remained consistently  below 35 ppb from 2001–2016 | 1.011 (1.008, 1.014) | 1.012 (1.011, 1.013) | 0.990 (0.989, 0.991) | 0.998 (0.991, 1.005) |

All models were adjusted for sex, race (Black/White/Other), Medicaid eligibility (yes/no), temperature, population density, % Black population, % American Indian and Alaska Native population, % Asian population, % Two or More Races population, % Native Hawaiian and Other Pacific Islander population, % Hispanic population, % population using automobile to transport, % population receiving less than high school education, % population above 65 years of age living below the poverty line, median household income, % population living in rented houses or apartments, distance to the nearest hospital, % of Medicare enrollees having at least one ambulatory visit to a primary care clinician in a year, % of diabetic Medicare enrollees aged 65–75 having hemoglobin A1c test in a year, number of hospitals, number of medical doctors, number of hospital beds, NDVI, BMI, and smoking rate. The models were also adjusted for other pollutants.

**Section 10. Pairwise correlation coefficients among 3-year average exposures to PM_2.5_ (μg/m^3^), NO_2_ (ppb), warm-season ozone (ppb), and PR (mBq/m^3^) in person-years with population density above or below 75th percentile**

| **Population density > 75th percentile** | | | | |
| --- | --- | --- | --- | --- |
|  | **PM_2.5_** | **NO_2_** | **Warm-season ozone** | **PR** |
| **PM_2.5_** | 1 | 0.51 | 0.29 | 0.48 |
| **NO_2_** |  | 1 | 0.26 | 0.23 |
| **Warm-season ozone** |  |  | 1 | 0.31 |
| **PR** |  |  |  | 1 |
|  | | | | |
| **Population density ≤ 75th percentile** | | | | |
|  | **PM_2.5_** | **NO_2_** | **Warm-season ozone** | **PR** |
| **PM_2.5_** | 1 | 0.33 | 0.31 | 0.48 |
| **NO_2_** |  | 1 | 0.34 | 0.20 |
| **Warm-season ozone** |  |  | 1 | 0.39 |
| **PR** |  |  |  | 1 |

**Section 11. Hazard ratios of lung cancer per unit increase in 3-year average PM_2.5_ (μg/m^3^), NO_2_ (ppb), warm-season ozone (ppb), and PR (mBq/m^3^) exposures in multi-pollutant model with and without adjustment for neighborhood-level covariates**

| **Model** | **PM_2.5_** | **NO_2_** | **Warm-season ozone** | **PR** |
| --- | --- | --- | --- | --- |
| Main analysis with adjustment for  neighborhood-level covariates ^a^ | 1.008 (1.005, 1.011) | 1.013 (1.012, 1.013) | 0.991 (0.990, 0.992) | 1.005 (0.999, 1.012) |
| Analysis without adjustment for  neighborhood-level covariates ^b^ | 1.023 (1.020, 1.025) | 1.005 (1.005, 1.006) | 0.989 (0.988, 0.990) | 1.030 (1.025, 1.036) |

­^a^ Main analysis model specification

­ ^b^ Model without adjustment for neighborhood-level covariates

**References**

1. Chronic Conditions Data Warehouse. Condition Categories. Accessed Feb 22, 2022. <https://www2.ccwdata.org/web/guest/condition-categories>
